# The Poor Man's Home.—II

**Published:** 1897-08-28

**Authors:** 


### Modern Sociology.

### THE POOR MAN'S HOME.—II.

When we note that the price of City land put the speculative builder out of the competition in providing houses for the poorer classes, we must add that this was very often due to the successful greed of the speculative landlord. The poor man's lodging, like the poor man's clothes, are very often the cast-offs of the rich. When fashion, health, or convenience led the well-to-do from the centre of the City to its West-end or suburbs, their houses, the roomy, substantial mansions of an age when the jerry-builder was not, were sometimes used as offices or transformed into warehouses, but often they were rented by some shrewd man who sub-let them in single rooms. In the larger rooms partitions were put up to make them into as many apartments as there were windows, and each room, each window, meant a family. Formerly the house was the home of a family; now it was a hive.

Can we imagine what it is for a whole family to live in one room—father, mother, sons and daughters of all ages, often kinsmen and women, sometimes even lodgers? Dr. Russell, the senior medical officer for Glasgow, told what it meant in an impassioned address delivered in 1888, which stirred the inhabitants of the Second City to even greater efforts in the cause of providing decent accommodation for its poor, efforts which have been so successful that many observers think that in this matter Glasgow has shown the way to the world. Dr. Russell told his hearers that these one-room houses had an average of three inmates, but thousands of them contained five, six, and seven inmates, and hundreds from eight up even to thirteen. "I might throw down that statement before you, and ask you to imagine yourselves, with all your appetites and passions, your bodily necessities and functions, your feelings of modesty, sense of propriety, your births, your sicknesses, your deaths, your children—in short, your *lives* in the whole round of their relationships with the seen and unseen, suddenly shrivelled and shrunk into such conditions of space. I might ask you, I do ask you, to consider and honestly confess what would be the result to you. But I would fain do more, generalities are so feeble. Yet, how can I speak to you decently of details? Where can I find language in which to clothe the facts of these poor people's lives, and yet be tolerable?"

And this, indeed, hampers all writers and speakers on the subject. We who have been brought up in an atmosphere of decency cannot bring ourselves to say plainly what are the conditions in these dens, and it needs their own inhabitants to tell the full unmitigated truth, to tell it simply and naturally, because they have no conception of reticence, because it seems to them that filth, promiscuity, drunkenness, riot, and immorality are the natural conditions of life. Yet some things Dr. Russell did tell his hearers. He told them that while in the districts where the houses were larger the death-rate was 16 or 17 per 1,000 of the population, among the small houses the death-rate was 38 per 1,000. He told them that one in every five children born in these poor districts dies before the end of its first year. He told them that of all the children who die in Glasgow before the completion of their first year, nearly a third die in these one-roomed houses—die of bad and insufficient air, of bad and insufficient food, of the careless handling of over-burdened mothers, whose work and worry often makes them indifferent and degraded.

And what is true of Glasgow is true of London, of Liverpool, of Dublin, of every great city in the Kingdom. If it be more true of Glasgow than of another place it is because Glasgow is, more than any other, the city of the one-roomed house. The census of 1881 showed that 25 per cent. of its inhabitants lived in one-roomed houses, and 45 per cent. occupied houses of two rooms. As it is certain that we cannot class so much as 70 per cent. of the inhabitants of Glasgow as the very poor, it is to be inferred that those feelings of modesty which we consider innate do not exist among a considerable number of people who could reasonably afford the conveniences they demand. Things have improved immensely since 1881, but it must be admitted that a sense of decency in lodging, a feeling of the propriety of separating the sexes, even among adults, is simply non-existent among the humbler classes in Scotland. In a block of houses recently built in a village not far from Glasgow it was found impossible to let houses of two rooms except to people who meant to take lodgers, and this although the rents were moderate. The proprietor reluctantly rearranged them as single-room houses, but provided only one bed in each. Still they did not let. The proprietor at length asked a man who had looked at the houses, hesitated, and at last refused to take one, what was the objection. The man admitted that they were well built, and convenient; that they had an advantage over many as high-rented in having as out-buildings a laundry with a good boiler, locked coal-cellars, decent and sanitary closets; that the site was healthy, the neighbourhood respectable. With what, then, did he find fault? The explanation was prompt; there was only one bed.

"But," said the proprietor, "you are a newly-married man; you have no children; why do you need another bed?"

"If a friend came to see us," was the immediate reply, "we might want another bed for him."

When at last the landlord gave up the attempt to improve the notions of the working classes in the matter of propriety, and put two beds in each room, the houses let at once.

In rural districts casual labourers required only at special seasons, such as reapers and potato-diggers, are still often housed in barns in absolute promiscuity, and there seems to be no compulsion to make their employers provide for them more decently.

It must be insisted on that it is not poverty alone that keeps people crowded into unhealthy dens without regard to propriety; it is a degraded idea of life which may have sprung originally from poverty, but is by no means immediately dependent on it. Many people pay as much for a bad house as they could get a good one for; especially if the occupancy of the good house be conditioned by any regulations as to over-crowding, propriety of conduct, and the like, they will have none of it. This, indeed, forms the opportunity of the unscrupulous landlord, or more often the tenant-in-chief, who takes a house and sub-lets. Sir Charles Cameron, in a memorandum on the subject, mentions a house in Dublin, the valuation rent of which was £18, but which was let to eleven families, whose combined rents amounted to £74 2s. Many of these tenants had but poor and uncertain incomes; but among them were a jeweller making £1 8s. a week, a bricklayer earning £1 12s., and two carpenters each receiving £1 14s. Unless these men dissipated their wages they could certainly have afforded a better home than one or two rooms in this tenement could give them.

